# Neuroendocrine differentiation in prostate neoplasms

**DOI:** 10.1007/s00292-025-01449-3

**Published:** 2025-08-25

**Authors:** Rainer Grobholz

**Affiliations:** 1 Medical Faculty, University of Zurich, Zurich, Switzerland; 2 Institute of Pathology, Cantonal Hospital Aarau, Tellstrasse 25, 5001 Aarau, Switzerland

**Keywords:** Adenocarcinoma, Androgen receptors, Cell transdifferentiation, Immunohistochemistry, Prognosis, Adenokarzinom, Androgenrezeptoren, Transdifferenzierung, Immunhistochemie, Prognose

## Abstract

Neuroendocrine (NE) cells in the prostate are part of the diffuse NE system and are found in both the normal prostate and acinar adenocarcinomas, occasionally exhibiting Paneth cell-like morphology.

NE cells produce peptide hormones and biogenic amines that influence the differentiation and growth of the prostate glands through paracrine signaling; however, they do not show proliferative activity and lack androgen receptors (AR).

Prostate tumors with NE differentiation are classified into five groups: (1) acinar adenocarcinomas with partial NE differentiation, detectable only by immunohistochemistry, (2) adenocarcinomas with Paneth cell-like differentiation, (3) NE tumors/carcinoids (NET), (4) small cell NE carcinomas (SCNEC), and (5) large cell NE carcinomas (LCNEC).

The significance of partial and Paneth cell-like differentiation in adenocarcinomas remains under discussion and plays a minor role in routine diagnostics. NETs are extremely rare and appear to behave similarly to NETs of the gastrointestinal tract. In contrast, SCNECs and LCNECs are aggressive tumors with important clinical relevance, as they have a poor prognosis and require aggressive treatment.

Therapy-associated neuroendocrine prostate carcinomas (t-NEPC) are recognized as a distinct entity for the first time in the WHO classification (5th edition, 2022). It arises through transdifferentiation via epigenetic changes following androgen deprivation and is characterized by AR loss and high proliferation, among other features. As with primary NE carcinomas, aggressive therapy is indicated. Therefore, a follow-up biopsy is recommended for castration-resistant progressive prostate cancer to confirm this aggressive phenotype.

## Abstract (de)

Neuroendokrine (NE) Zellen in der Prostata gehören zum diffusen neuroendokrinen System und finden sich in der normalen Prostata und in azinären Adenokarzinomen, teilweise auch mit Paneth-Zell-ähnlicher Morphologie.

NE-Zellen produzieren Peptidhormone und biogene Amine, die parakrin die Differenzierung und das Wachstum der Prostatadrüsen beeinflussen. Sie zeigen jedoch keine proliferative Aktivität und besitzen keinen Androgenrezeptor (AR).

Prostatatumoren mit NE-Differenzierung werden in 5 Gruppen eingeteilt: (1) azinäre Adenokarzinome mit partieller, nur immunhistochemisch erkennbarer NE-Differenzierung, (2) Adenokarzinome mit Paneth-Zell-ähnlicher Differenzierung, (3) NE-Tumoren/Karzinoide (NET), (4) kleinzellige NE-Karzinome (SCNEC) und (5) großzellige NE-Karzinome (LCNEC).

Die Bedeutung partieller und Paneth-Zell-ähnlicher Differenzierung in Adenokarzinomen wird diskutiert und spielt im diagnostischen Alltag eine untergeordnete Rolle. NET sind äußerst selten und scheinen sich wie NET des Gastrointestinaltraktes zu verhalten. Die SCNEC und LCNEC sind hingegen aggressive Tumoren mit wichtiger klinischer Bedeutung, da sie eine schlechte Prognose besitzen und eine aggressive Therapie nötig ist.

Das therapieassoziierte neuroendokrine Prostatakarzinom (t-NEPC) ist in der 5. Auflage der WHO-Klassifikation (2022) erstmals eine eigene Entität. Es entsteht durch Transdifferenzierung via epigenetischer Veränderungen nach Androgenentzug und weist u. a. einen AR-Verlust und eine hohe Proliferation auf. Wie bei den primären neuroendokrinen Karzinomen ist eine aggressive Therapie indiziert. Daher ist bei kastrationsresistenten progredienten Prostatakarzinomen eine Verlaufsbiopsie zum Nachweis dieses aggressiven Phänotyps zu empfehlen.

## Introduction

Neuroendocrine (NE) cells in the prostate were first described by Pretl in 1944, and even then, it was assumed that these cells belong to the diffuse neuroendocrine system rather than the actual glandular prostate tissue [1]. This assumption was later confirmed by Aumüller and colleagues, who demonstrated that the NE cells of the prostate are of neurogenic origin and, alongside basal cells and secretory cells, constitute a distinct cell population that migrates into the prostate during embryonic development. Accordingly, specific prostate markers cannot be found in NE cells [2]. In contrast, other studies have identified co-expression of NE and prostate markers, leading to the concept of a stem cell model for the presence and development of NE cells in the prostate [3].

Neuroendocrine cells are distributed with varying frequency across all regions of the prostate and can produce various peptide hormones and biogenic amines, which influence the differentiation and growth regulation of the prostate glands through paracrine mechanisms [4]. Neuroendocrine cells in the prostate show no proliferative activity, lack androgen receptors (AR), and are therefore hormone-insensitive [3].

Prostate tumors with NE differentiation are pathologically classified into five groups [5]:

1. Acinar adenocarcinomas with NE differentiation.
2. Adenocarcinomas with Paneth cell-like differentiation.
3. Neuroendocrine tumors/carcinoids.
4. Small cell NE carcinomas (SCNEC).
5. Large cell NE carcinomas (LCNEC).

In the new World Health Organization (WHO) classification (fifth edition, 2022), treatment-associated neuroendocrine prostate carcinoma (t-NEPC) is listed as a distinct entity in the chapter “Tumors of the prostate.” All other NE tumors are categorized in a separate chapter entitled “Neuroendocrine neoplasms” for all urogenital organs.

## Acinar adenocarcinomas with partial NE differentiation

These are tumors with the morphology of an acinar or ductal carcinoma containing neuroendocrine tumor cells (NETC), which can only be detected through immunohistochemical staining for NE markers (chromogranin A or synaptophysin).

The presence of NETC in acinar adenocarcinomas of the prostate is a well-known phenomenon. Several studies have shown that an increased intracellular cyclic adenosine monophosphate (cAMP) level promotes the transdifferentiation of exocrine tumor cells into NETC in vitro [6]. During this transdifferentiation, tumor cells lose AR expression and no longer exhibit proliferative activity. This effect can be achieved, among other methods, through androgen deprivation and is reversible upon the addition of androgen-containing medium [7, 8], thereby highlighting the plasticity of these tumor cells. In vivo, microdissection and allele sequencing of NETC and exocrine tumor cells have demonstrated that both cell types show an identical profile, suggesting that the process of transdifferentiation also occurs in vivo [8].

**Fig. 1:**
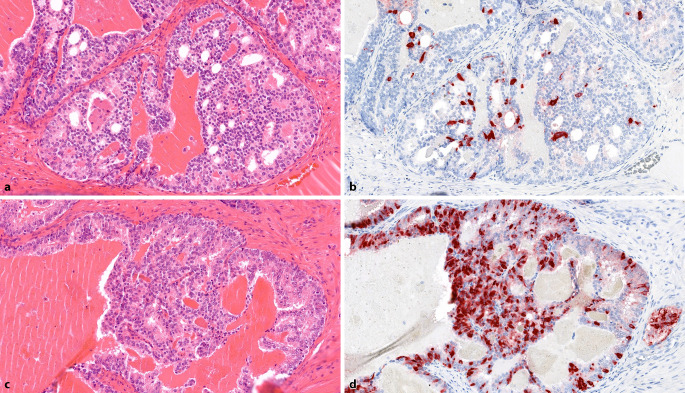
Prostate adenocarcinoma with partial neuroendocrine differentiation. **a** Acinar adenocarcinoma without evident neuroendocrine differentiation (H&E, ×200). **b** Serial section of **a**: Single, interspersed neuroendocrine tumor cells (chromogranin A, ×200). **c** Acinar adenocarcinoma without evident neuroendocrine differentiation (H&E, ×200). **d** Serial section of **c**: numerous neuroendocrine tumor cells, some arranged in groups (chromogranin A, ×200)

The extent of NETC in untreated prostate carcinomas (PCa) varies considerably, ranging from a few scattered NETC to large clusters (Fig. 1a–d; [9]). Neuroendocrine tumor cells in PCa are specialized non-proliferative AR-negative cells that produce and secrete bioactive molecules such as serotonin and neuropeptides. These bioactive substances can affect neighboring tumor cells and the microenvironment, promoting their growth and angiogenesis [10, 11].

Consistent and reproducible quantification—and thus determination of the extent of NETC—is challenging and handled differently across studies. Therefore, it is not surprising that there are varying results regarding the clinical significance of partial NE differentiation in PCa. Most studies have found a correlation between the extent of NE differentiation and the Gleason score (with more NETC observed in tumors with higher Gleason scores); however, concerning biochemical recurrence and overall survival, significance was only found in univariate analyses, not in multivariate ones.

Due to the lack of clinical significance and therapeutic consequences of partial NE differentiation in acinar PCa, this phenomenon is no longer listed as a separate category in the fifth edition of the WHO classification. *Routine testing for NE markers (e.g., chromogranin A or synaptophysin) is not recommended for untreated acinar PCa.*

## Prostate carcinomas with Paneth cell-like NE differentiation

**Fig. 2:**
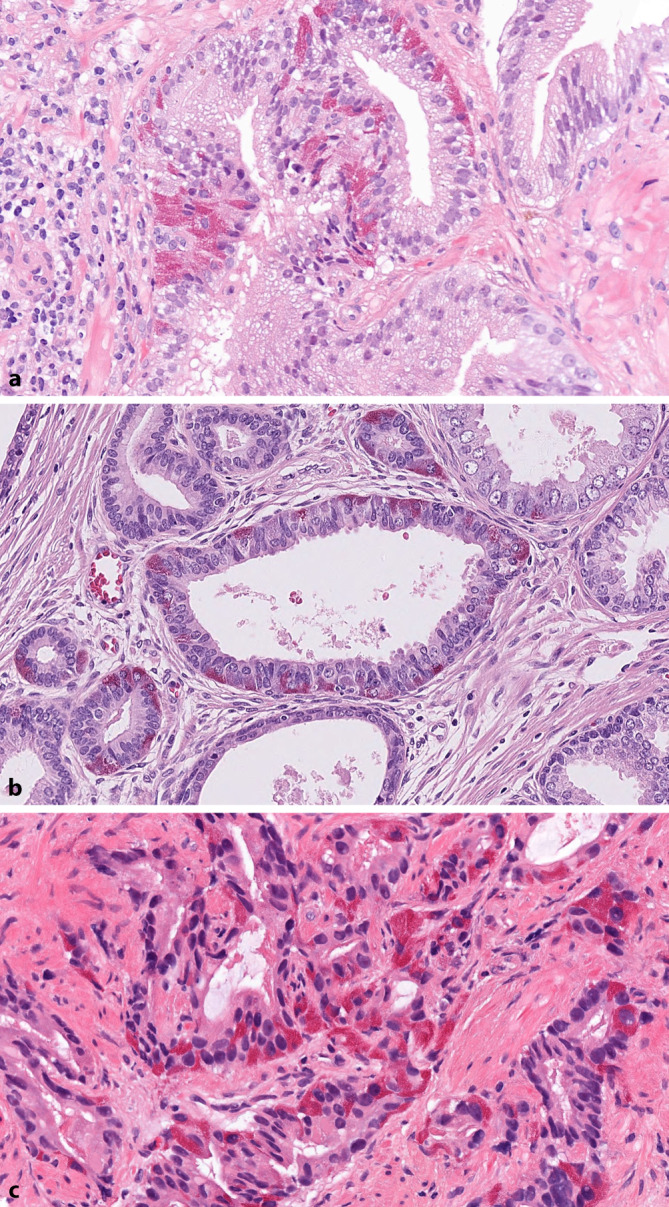
Paneth cell differentiation with intense, finely granular eosinophilic cytoplasm in non-tumorous prostate glands (**a**; H&E, ×400), in high-grade prostatic intraepithelial neoplasia (**b**; H&E, ×400), and in invasive acinar adenocarcinoma (**c**; H&E, ×400)

Paneth cell-like NE differentiation in the prostate is occasionally found in normal epithelium (Fig. 2a) as well as in high-grade prostatic intraepithelial neoplasia (Fig. 2b) and adenocarcinomas (Fig. 2c; [12]). These cells are characterized by intense, finely granular eosinophilic cytoplasm, which is immunohistochemically positive for NE markers and negative for lysozyme and IgA, in contrast to Paneth cells of the small intestine [12]. As these tumor cells are easily recognizable in hematoxylin/eosin-stained sections, immunohistochemical staining is not necessary.

Paneth cell-like NE differentiation in prostatic adenocarcinoma may occur either as isolated, scattered cells or diffusely within glands or nests. These Paneth cell-like cells can be present in well-formed glands of Gleason pattern 3, but they may also appear exclusively in cell strands with inconspicuous cytology, which, according to strict application of the Gleason grading system, would be assigned a Gleason pattern 5.

In three series totaling 127 cases, it was shown that patient outcomes were primarily linked to the Gleason score of the tumor areas without Paneth cell-like differentiation [13–15]. Incorporating the Paneth cell-like areas into the Gleason grading led to significant upgrading, which no longer correlated with patient outcomes.

For this reason, it is recommended that grading in such cases should only consider the areas of acinar (and/or ductal) carcinoma, excluding the regions with Paneth cell-like differentiation [13–15]. This is especially important when Paneth cell-like areas within an otherwise low-grade tumor environment would result in upgrading to a high-grade tumor.

## Neuroendocrine tumors/carcinoids

True neuroendocrine tumors (NET) of the prostate are extremely rare. To diagnose a NET of the prostate and distinguish it from a PCa with carcinoid-like features, the following criteria should be met: (1) not directly associated with a concurrent acinar adenocarcinoma of the prostate, (2) immunohistochemically positive for NE markers and negative for prostate-specific antigen (PSA), and (3) originating from the prostatic parenchyma.

Applying these criteria, seven cases have been documented in the literature. Three cases involved patients with multiple endocrine neoplasia type IIB at the ages of 7, 19, and 22 years [16, 17]; three cases involved patients aged 33, 34, and 37 years [18, 19]; and one recently published case described a 67-year-old patient who presented with a separate 1‑mm NET in addition to an acinar PCa [20].

Due to the small number of cases, it is challenging to predict the clinical and biological course of these tumors in the prostate. Most of the documented cases presented at a locally advanced stage, with one case involving lymph node metastases. Nevertheless, all cases showed a favorable prognosis, suggesting that grading these tumors—in analogy to NET of the gastrointestinal tract—based on mitotic count and proliferation rate (Ki67) is appropriate. Grading according to the Gleason score does not apply to NET.

In contrast, it is important to differentiate tumors with a carcinoid-like morphology that are associated with an ordinary acinar or ductal adenocarcinoma. In these areas, the tumor cells are arranged in organoid nests or islands, resembling a NET. However, the tumor cell nuclei often retain the characteristic prominent nucleolus typical of prostate carcinomas, with only occasional finely granular chromatin.

To further distinguish these, prostate-specific markers (e.g., PSA) should be used, as true NETs are negative for PSA, while ordinary carcinomas with carcinoid-like morphology are PSA positive. Immunohistochemical staining for prostate-specific acid phosphatase (PSAP) is not recommended for differentiation, as PSAP can also be expressed in non-prostatic NET [21, 22].

Due to the rarity of true NETs, there are still insufficient data regarding the expression of other prostate-specific markers such as NKX3.1, prostein (P501S), and others. Tumors with carcinoid-like morphology are graded like ordinary PCa using the Gleason score.

## Small cell carcinomas

**Fig. 3:**
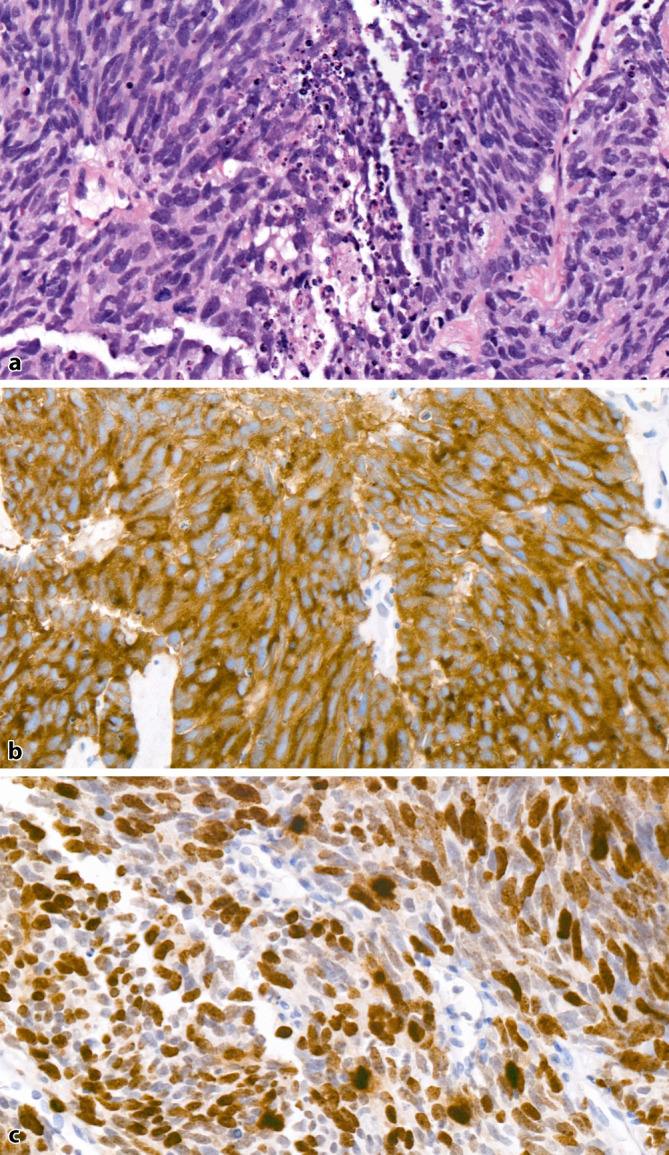
**a** Small cell carcinoma of the prostate with scant cytoplasm and dark condensed chromatin without prominent nucleoli. Numerous mitotic figures and apoptotic bodies with central necrotic debris (H&E, ×400). **b** Strong, consistent expression of synaptophysin in the tumor cells (×400). **c** High proliferation rate of approximately 70–80% (Ki67, ×400)

Small cell carcinomas (SCNECs) are high-grade tumors with a characteristic morphology similar to small cell carcinomas of the lung. At low magnification, the tumor tissue appears blue and densely cellular, with small tumor cells exhibiting scant cytoplasm, nuclear molding, frequent nuclear smearing artifacts, and dark condensed chromatin without prominent nucleoli. The tumors have a high mitotic rate and numerous apoptotic figures and are commonly observed with tumor necrosis (Fig. 3a).

As with lung tumors, an intermediate cell type with less dense chromatin and small but visible nucleoli can also appear as a morphological variant. Immunohistochemically, the tumors are positive for at least one NE marker—synaptophysin, chromogranin A, or CD56—in more than 90% of cases (Fig. 3b). If NE markers are negative despite typical morphology, the detection of FOXA2 expression or the loss of RB1 expression can be helpful. FOXA2 expression is found in 75% of SCNECs of the prostate [23], and RB1 loss is observed in 90% of cases [24]. The proliferation rate (Ki67) is typically > 50%, often exceeding 80% (Fig. 3c). Grading according to the Gleason score is not performed for these tumors.

Primary pure SCNECs of the prostate are rare, with a frequency of < 1% and an incidence of 0.13–0.3 per 1,000,000 men [25–27]. The etiology of prostatic SCNEC remains unclear, but molecular data suggest that these tumors, even without prior treatment, arise through transdifferentiation from ordinary prostate carcinomas and may even share a common precursor cell [28–30].

**Fig. 4:**
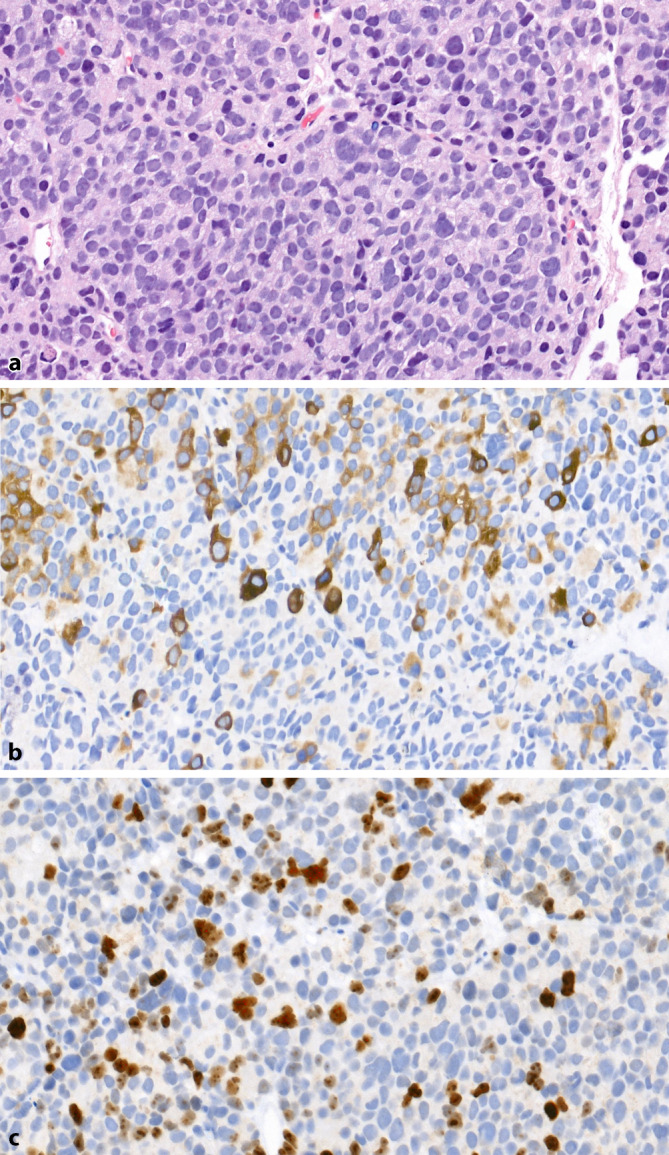
Poorly differentiated prostate adenocarcinoma, Gleason score 5 + 5. **a** Solid tumor growth; only a few tumor cell nuclei are enlarged with prominent nucleoli. **b** Neuroendocrine differentiation is only found in single cells, while most tumor cells are negative (synaptophysin, ×400). **c** Proliferation rate of approximately 20% (Ki67, ×400)

Distinguishing SCNEC from a poorly differentiated adenocarcinoma with solid growth (Gleason score 5 + 5) can be challenging, as the latter can also express NE markers, albeit in a patchy rather than a diffuse pattern (Fig. 4a, b). Unlike adenocarcinomas, SCNECs are immunohistochemically usually negative for prostate markers such as PSA, NKX3.1, or prostein. Expression of these markers is found in only about 17–25% of SCNEC cases, and even then, only in a few cells or small cell clusters. A diffuse expression of prostate markers across all tumor cells essentially excludes SCNEC.

As poorly differentiated adenocarcinomas typically have a proliferation rate of < 50%, Ki67 can also be useful (Fig. 4c). TTF‑1 is positive in approximately 50% of SCNECs, aiding in the differentiation from adenocarcinomas when positive. ERG expression, indicating a *TMPRSS2-ERG* fusion, is found in about 50% of SCNECs, which does not help to distinguish them from adenocarcinomas, as both show similar positivity rates.

As AR can also be expressed in SCNEC, AR expression is of only limited use in differentiation [31]. However, complete loss of AR is significant, suggesting a potential lack of response to antiandrogen therapy.

Localized SCNECs are typically treated aggressively, often with multimodal therapy consisting of chemotherapy and radiation. Metastatic SCNECs are managed with platinum-based combination chemotherapy, similar to small cell lung carcinomas. Some centers treat pure SCNECs solely with chemotherapy, while others also include antiandrogen therapy [32].

Patients with this aggressive disease frequently present with visceral metastases and less commonly with paraneoplastic syndromes. The median survival time is approximately 10 months, 13 months for those without metastases and 8 months for those with metastases [27].

## Treatment-associated neuroendocrine carcinomas

Treatment-associated neuroendocrine carcinomas (t-NEPCs) are tumors with complete or partial NE differentiation, usually arising in castration-resistant adenocarcinomas following androgen deprivation therapy. As previously mentioned, androgen deprivation can induce transdifferentiation into an NE phenotype. On a molecular level, inactivation (through mutation or inhibition) of *RB1 *and *TP53* has been identified as a key factor in this transdifferentiation. This dual inactivation triggers the reprogramming from an exocrine to an NE phenotype [33]. The inactivation of just one of these two genes alone, however, is not sufficient to induce transdifferentiation. Additionally, point mutations and amplifications of the *AR *gene can be observed, which, together with NE differentiation, contribute to AR loss [30]. Epigenetic changes also play a role in driving transdifferentiation: t‑NEPCs show hypermethylation in the promoter regions of various genes, including enhancer of zeste homolog 2 (*EZH2*), an enzyme that modifies histones, effectively silencing entire gene regions; t‑NEPCs exhibit twice the level of *EZH2 *expression compared to the remaining adenocarcinoma [30]. This is particularly relevant, as EZH2 inhibitors are already in clinical trials [34], offering the potential for a new targeted therapy. In the fifth edition of the WHO classification, t‑NEPC is listed as a distinct entity with its own chapter for the first time. More than half of these tumors develop within the first 24 months after androgen deprivation therapy, with a median survival time of 7 months [35].

**Fig. 5:**
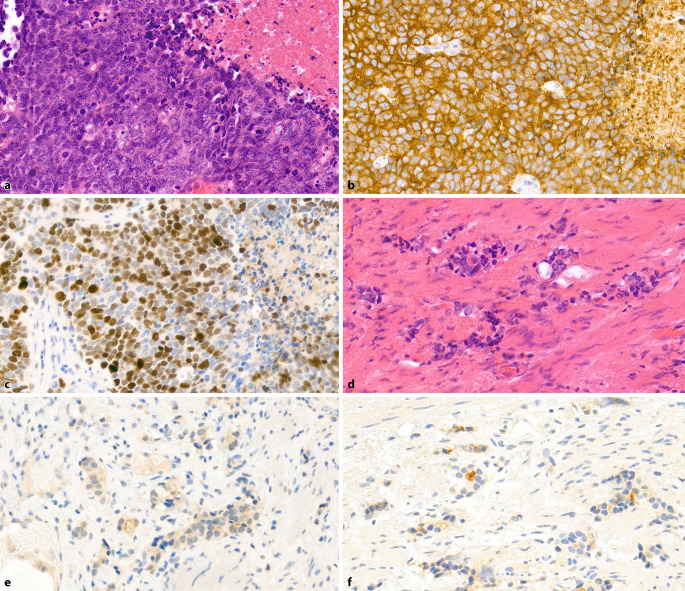
Treatment-associated neuroendocrine prostate carcinoma. **a** Morphology resembling small cell carcinoma with numerous mitotic figures and areas of necrosis (H&E, ×400). **b** Consistent expression of synaptophysin (×400). **c** High proliferation rate of approximately 70% (Ki67, ×400). **d** Adjacent regressive changes in pre-existing adenocarcinoma, with partially pyknotic cell nuclei and narrow cytoplasm (H&E, ×400). **e** Absence of basal cell keratin expression (CK34βE12, ×400). **f** Patchy expression of prostate-specific antigen (PSA, ×400)

When performing (palliative) transurethral resections or biopsies of metastases, clinicians often ask about “anaplasia” or “dedifferentiation.” These outdated terms are still used in clinical practice, and clinicians usually refer to the possible presence of a t-NEPC. Histologically, these tumors show a pattern similar to SCNECs and are therefore nearly indistinguishable (Fig. 5a–c). In most cases, remnants of pretreated adenocarcinoma with corresponding regressive changes are present, providing crucial diagnostic clues (Fig. 5d–f). The tumors are generally sharply demarcated from each other, although overlapping tumor areas can occasionally be seen. Gleason grading is not applied to t‑NEPC. Furthermore, the remaining portion of the pre-existing adenocarcinoma is also not graded, as treatment-induced regressive changes affect the architecture of the tumor glands. The biological and prognostic significance of this apparent dedifferentiation following androgen deprivation remains unclear, which is why Gleason grading is not performed [36].

In clinical practice, information about prior therapy is essential and crucial for diagnosis. If clinical information is lacking, identifying regressive changes in the pre-existing prostate tissue can serve as an indicator of prior treatment. In cases of uncertainty, it is recommended to include a comment noting the presumed prior treatment and explaining that Gleason grading was not performed as a result. Should the clinician confirm that no prior treatment took place, Gleason grading can be performed retrospectively.

## Large cell neuroendocrine carcinoma

**Fig. 6:**
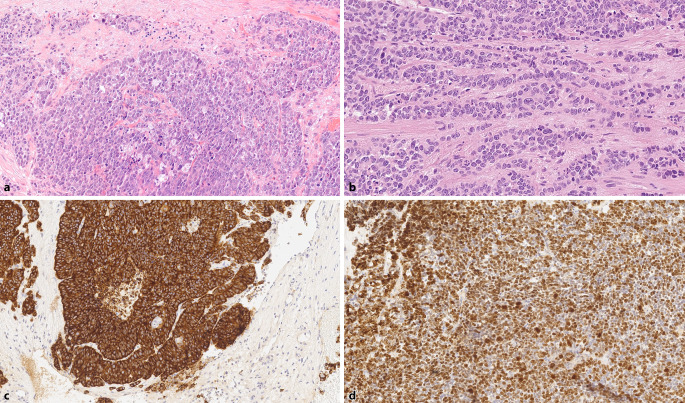
Large cell neuroendocrine prostate carcinoma. **a** Solid nests of tumor cells with peripheral palisading (H&E, ×200). **b** Strand-like growth pattern, nuclei with finely granular chromatin and small nucleoli (H&E, ×400). **c** Consistent expression of synaptophysin (×200). **d** High proliferation rate > 90% (Ki67, ×200)

Large cell neuroendocrine carcinomas (LCNECs) are high-grade tumors characterized by large sheets of tumor cells with occasional peripheral palisading, tumor cells arranged in strands, and often central necrosis (Fig. 6a). The tumor cells display large nuclei with open chromatin, sometimes visible nucleoli, and well-defined cytoplasm (Fig. 6b). Mitotic figures are frequently observed.

Immunohistochemically, LCNECs show consistent expression of at least one NE marker (chromogranin A, synaptophysin, or CD 56), and the proliferation rate (Ki67) exceeds 50% (Fig. 6c, d). Prostate markers such as PSA and PSAP can be expressed, and in some cases, AR expression is also present [37]. LCNECs are rare tumors, with approximately 25 cases described in the literature—13 primary cases without prior antiandrogen therapy and 12 cases following antiandrogen treatment [38]. Due to the rarity of this tumor, extensive data on their expression profiles are lacking. The treatment approach mirrors that of SCNEC. The prognosis is poor, with the largest case series reporting a median survival time of 7 months [39]. As with SCNEC, it is crucial to distinguish LCNEC from a poorly differentiated, solid-growing prostate carcinoma (Gleason score 5 + 5), as the prognosis and treatment for these two tumor types differ significantly. The same distinguishing features described for SCNEC also apply here. In general, for poorly differentiated PCa with solid growth and no clear acinar differentiation, the prostatic origin should be confirmed—or, alternatively, SCNEC/LCNEC or a tumor of non-prostatic origin must be ruled out.

## Practical conclusion

- Neuroendocrine (NE) differentiation in prostate carcinoma (PCa) ranges from minimal, clinically insignificant forms to aggressive NE carcinomas.
- Acinar adenocarcinomas with partial NE differentiation are no longer listed separately due to their lack of clinical and biological significance.
- Neuroendocrine tumors/carcinoids are extremely rare in the prostate and are subject to strict diagnostic criteria.
- Small cell and large cell neuroendocrine carcinomas must be recognized and diagnosed due to their distinct treatment strategies.
- In the fifth edition of the WHO classification, treatment-associated neuroendocrine prostate carcinoma (t-NEPC) is listed as a distinct entity for the first time.
- All other NE tumors of the prostate are categorized in a separate chapter covering all urogenital organs.
- t‑NEPC arise after hormone deprivation therapy in castration-resistant PCa and represent highly aggressive tumors.
- Differentiation between aggressive neuroendocrine carcinomas and poorly differentiated non-neuroendocrine PCa relies on lineage-specific immunohistochemical markers and proliferation rates.

## Declarations

For this article no studies with human participants or animals were performed by any of the authors. All studies mentioned were in accordance with the ethical standards indicated in each case.

The supplement containing this article is not sponsored by industry.
